# Correction: A unicellular relative of animals generates a layer of polarized cells by actomyosin-dependent cellularization

**DOI:** 10.7554/eLife.60055

**Published:** 2020-06-19

**Authors:** Omaya Dudin, Andrej Ondracka, Xavier Grau-Bové, Arthur AB Haraldsen, Atsushi Toyoda, Hiroshi Suga, Jon Bråte, Iñaki Ruiz-Trillo

Dudin O, Ondracka A, Grau-Bové X, Haraldsen AAB, Toyoda A, Suga H, Bråte J, Ruiz-Trillo I. 2019. A unicellular relative of animals generates a layer of polarized cells by actomyosin-dependent cellularization. *eLife* **8**:e49801. doi: 10.7554/eLife.49801.

Published 24, October 2019

We have identified an error in the reported genome size of *S. arctica*. This error was unintentional and reported the genome size of a previous and older version of the genome. This error was found twice in the manuscript. See below.

[1] Under heading- ‘Cellularization is associated with extensive sequential transcriptional waves and is associated with evolutionarily younger transcripts’:

“The final assembly sequences comprised 115,261,641 bp, and the metrics were greatly improved compared to the previous assembly”

The assembly size is 142,721,209 bp.

[2] Figure 3—source data 1:

“Total sequence length of Version 2.1 (this study): 115,261,641 bp”

The assembly size is 142,721,209 bp.

The article has been corrected accordingly.

