# Supplementary material for: A unicellular relative of animals generates a layer of polarized cells by actomyosin-dependent cellularization
Source: eLife. 2019 Oct 28;8:e49801. doi: 10.7554/eLife.49801 (PMC6855841; doi:10.7554/eLife.49801)
Supplement: Figure 3—source data 1. [file elife-49801-fig3-data1.pdf]

Table 1

Metrics of the *S. arctica* genome assemblies

Metrics	Version 1 (Grau-Bove et al. 2017)	Version 2.1 (this study)
Total sequence length	121,588,341 bp	142,721,209 bp
Number of supercontigs	15,618	737
Supercontig N50	64,598 bp	243,943 bp
Longest supercontig	357,797 bp	776,519 bp

Metrics of the *S. arctica* genome assemblies (without the mitochondrial genome)

Metrics	Version 1 (Grau-Bove et al. 2017)	Version 2.1 (this study)
Total sequence length	121,588,341 bp	115,079,836 bp
Number of supercontigs	15,618	732
Supercontig N50	64,598 bp	244,210 bp
Longest supercontig	357,797 bp	776,519 bp
