# Supplementary material for: A unicellular relative of animals generates a layer of polarized cells by actomyosin-dependent cellularization
Source: eLife. 2019 Oct 28;8:e49801. doi: 10.7554/eLife.49801 (PMC6855841; doi:10.7554/eLife.49801)
Supplement: Figure 4—source data 2. [file elife-49801-fig4-data2.pdf]

Figure 4-source data 2
List of genomes and transcriptomes used in the Blastn search to detect conserved S. arctica lncRNAs. SRA indicates the accession number to raw data available from the NCBI Short Read Archive. “Trinity de novo” indicates that the transcriptome was assembled without a reference genome. “Trinity de novo + ref + PASA genome” indicates that the transcriptome was assembled using a reference genome. Assembly details are described in the methods.

Species	**Transcriptome accessions (assembly strategy)**	**Genome accessions**
Sphaeroforma sirkka	SRA ERR2729814 (Trinity de novo)	
Sphaeroforma napiecek	SRA ERR2709822 (Trinity de novo)	
Sphaeroforma gastrica	Unpublished RNA-seq (Trinity de novo + ref + PASA genome)	
Sphaeroforma tapetis	Unpublished RNA-seq (Trinity de novo + ref + PASA genome)	
Sphaeroforma nootkatensis	Unpublished RNA-seq (Trinity de novo)	
Creolimax fragrantissima	SRA ERR2666850 (Trinity de novo)	
Abeoforma whisleri	Unpublished RNA-seq (Trinity de novo)	
Pirum gemmata	Unpublished RNA-seq (Trinity de novo)	
Amoebidium parasiticum	SRA SRR343044, SRR343049, SRR545192 (Trinity de novo)	
Chromosphaera perkinsii	SRA SRR5192512 (Trinity de novo)	
Ichthyophonus hoferi	SRA SRR1618711 (Trinity de novo)	
Sphaerotecum destruens	SRA SRR1618479 (Trinity de novo)	
Ministeria vibrans	SRA SRR343051, SRR343053 (Trinity de novo)	
Capsaspora owczarzaki	GCA_000151315.2	GCA_000151315.2
Salpingoeca rosetta	GCF_000188695.1	GCF_000188695.1
Monosiga brevicolis	GCF_000002865.3	GCF_000002865.3
Didymoeca costata	SRA SRR6344966 (Trinity de novo)	
Choanoeca perplexa	SRA SRR6344967 (Trinity de novo)	
Salpingoeca infusionum	SRA SRR6344968 (Trinity de novo)	
Microstomoeca roanoka	SRA SRR6344969 (Trinity de novo)	
Diaphanoeca grandis	SRA SRR6344970 (Trinity de novo)	
Acanthoeca spectabilis	SRA SRR6344971 (Trinity de novo)	
Salpingoeca dolichothecata	SRA SRR6344972 (Trinity de novo)	
Codosiga hollandica	SRA SRR6344973 (Trinity de novo)	
Salpingoeca helianthica	SRA SRR6344974 (Trinity de novo)	
Mylnosiga fluctuans	SRA SRR6344975 (Trinity de novo)	
Salpingoeca punica	SRA SRR6344976 (Trinity de novo)	
Hartaetosiga gracilis	SRA SRR6344977 (Trinity de novo)	
Salpingoeca kvevrii	SRA SRR6344978 (Trinity de novo)	
Salpingoeca urceolata	SRA SRR6344979 (Trinity de novo)	
Stephanoeca diplocostata	SRA SRR6344980 (Trinity de novo)	
Helgoeca nana	SRA SRR6344981 (Trinity de novo)	
Stephanoeca diplocostata	SRA SRR6344982 (Trinity de novo)	
Savillea parva	SRA SRR6344983 (Trinity de novo)	
Salpingoeca macrocollata	SRA SRR6344984 (Trinity de novo)	
Hartaetosiga balthica	SRA SRR6344985 (Trinity de novo)	
