# Supplementary material for: A unicellular relative of animals generates a layer of polarized cells by actomyosin-dependent cellularization
Source: eLife. 2019 Oct 28;8:e49801. doi: 10.7554/eLife.49801 (PMC6855841; doi:10.7554/eLife.49801)
Supplement: Figure 4—source data 4. [file elife-49801-fig4-data4.pdf]

Sheet1

Species name	Short name	Source
H. sapiens	Hsap	Ensembl 80
S. kowalewskii	Skow	
D. melanogaster	Dmel	Ensembl Metazoa 27
N. vectensis	Nvec	Ensembl Metazoa 27
T. adherens	Tadh	Ensembl Metazoa 27
M. leidii	Mlei	Ensembl Metazoa 27
A. queenslandica	Aque	Ensembl Metazoa 27
S. rosetta	Sros	Ensembl Protist 27
C. owczarczaki	Cowc	https://figshare.com/articles/Genome_-_Capsaspora_owczarzaki_v3_/4123158
M. vibrans	Mvib	
S. arctica	Sarc	this study
C. fragrantissima	Cfra	https://figshare.com/articles/Creolimax_fragrantissima_genome_data/1403592
I. Hoferi	Ihof	https://figshare.com/articles/Genome_-_Ichthyophonus_hoferi/5426488
A. whisleri	Awhi	https://figshare.com/articles/Genome_-_Abeoforma_whisleri_/5426458
C. perkinsii	Cper	https://figshare.com/articles/Genome_-_Chromosphaera_perkinsii/5426494
P. gemmata	Pgem	https://figshare.com/articles/Genome_-_Pirum_gemmata/5426506
S. destruens	Sdes	https://figshare.com/articles/Transcriptome_-_Sphaerothecum_destruens_rosette_agent_/5446489
C. limatocisporum	Clim	https://figshare.com/articles/Genome_-_Corallochytrium_limacisporum/5426470
F. alba	Falb	SRP022580
C. anguillulae	Cang	Ensembl Fungi 27
S. punctatus	Spun	SRR343043
M. verticillata	Mver	
S. pombe	Spom	Ensembl Fungi 27
T. trahens	Ttra	
A. castellanii	Acas	Ensembl Protist 27
D. discoideum	Ddis	Ensembl Protist 27
N. gruberi	Ngru	Ensembl Protist 27
T. thermophila	Tthe	Ensembl Protist 27
E. huxleyi	Ehux	Ensembl Protist 27
A. thaliana	Atha	Ensembl Plants 27
P. yezoensis	Pyez	
