# Supplementary material for: A unicellular relative of animals generates a layer of polarized cells by actomyosin-dependent cellularization
Source: eLife. 2019 Oct 28;8:e49801. doi: 10.7554/eLife.49801 (PMC6855841; doi:10.7554/eLife.49801)
Supplement: Figure 5—source data 1. [file elife-49801-fig5-data1.pdf]

Figure 5-source data 1

Gene ontology enrichement of expression clusters

GO: ID	GO Term	p value
	**Cluster 1**	
GO:0040010	positive regulation of growth rate	2.5e-19
GO:0040035	hermaphrodite genitalia development	1.5e-13
GO:0006364	rRNA processing	6.5e-12
GO:0002119	nematode larval development	2.3e-11
GO:0006898	receptor-mediated endocytosis	1.4e-09
GO:0000398	mRNA splicing, via spliceosome	7.2e-07
GO:0006259	DNA metabolic process	1.5e-06
GO:0009792	embryo development ending in birth or egg hatching	2.6e-06
GO:0042254	ribosome biogenesis	8,00E-06
GO:0000381	regulation of alternative mRNA splicing, via spliceosome	8.4e-06
GO:0006626	protein targeting to mitochondrion	1.6e-05
GO:0030174	regulation of DNA-dependent DNA replication initiation	3.5e-05
GO:0030261	chromosome condensation	4.9e-05
GO:0040020	regulation of meiotic nuclear division	6.5e-05
GO:0051084	de novo' posttranslational protein folding	7.1e-05
GO:0022008	neurogenesis	8.5e-05
GO:0032201	telomere maintenance via semi-conservative replication	9.4e-05
GO:0006271	DNA strand elongation involved in DNA replication	1,00E-04
GO:0006412	translation	0.00016
GO:0006385	transcription elongation from RNA polymerase III promoter	0.00048
GO:0006386	termination of RNA polymerase III transcription	0.00048
GO:0017183	peptidyl-diphthamide biosynthetic process from peptidyl-histidine	0.00048
GO:0030488	tRNA methylation	0.00053
GO:0031167	rRNA methylation	0.00059
GO:0010833	telomere maintenance via telomere lengthening	0.00064
GO:0090305	nucleic acid phosphodiester bond hydrolysis	0.00065
GO:0006997	nucleus organization	0.00068
GO:0000731	DNA synthesis involved in DNA repair	0.00114
GO:0006418	tRNA aminoacylation for protein translation	0.00163
GO:0006369	termination of RNA polymerase II transcription	0.00174
GO:0016973	poly(A)+ mRNA export from nucleus	0.00193
GO:0006376	mRNA splice site selection	0.00193
GO:0006334	nucleosome assembly	0.00216
GO:0000466	maturation of 5.8S rRNA from tricistronic rRNA transcript (SSU-rRNA, 5.8S rRNA, LSU-rRNA)	0.00217
GO:0018992	germ-line sex determination	0.00217
GO:0019509	L-methionine salvage from methylthioadenosine	0.00221
GO:0009926	auxin polar transport	0.00221
GO:0007339	binding of sperm to zona pellucida	0.00221
GO:0009304	tRNA transcription	0.00221
GO:0006610	ribosomal protein import into nucleus	0.00221
GO:0043558	regulation of translational initiation in response to stress	0.00236
GO:0034969	histone arginine methylation	0.00236
GO:0009303	rRNA transcription	0.00267
GO:0022618	ribonucleoprotein complex assembly	0.00279
GO:0006406	mRNA export from nucleus	0.00293

**GO:0000722**	telomere maintenance via recombination	0.00297
**GO:0030490**	maturation of SSU-rRNA	0.00312
**GO:0006595**	polyamine metabolic process	0.00321
**GO:0009113**	purine nucleobase biosynthetic process	0.00321
**GO:0032259**	methylation	0.00404
**GO:0044260**	cellular macromolecule metabolic process	0.00408
**GO:0048024**	regulation of mRNA splicing, via spliceosome	0.00443
**GO:0043928**	exonucleolytic catabolism of deadenylated mRNA	0.00459
**GO:0006268**	DNA unwinding involved in DNA replication	0.00472
**GO:0044743**	protein transmembrane import into intracellular organelle	0.00481
**GO:0034660**	ncRNA metabolic process	0.00572
**GO:0007076**	mitotic chromosome condensation	0.00628
**GO:0002183**	cytoplasmic translational initiation	0.00677
**GO:0042246**	tissue regeneration	0.00677
**GO:0000480**	endonucleolytic cleavage in 5'-ETS of tricistronic rRNA transcript (SSU-rRNA, 5.8S rRNA, LSU-rRNA)	0.00677
**GO:0000472**	endonucleolytic cleavage to generate mature 5'-end of SSU-rRNA from (SSU-rRNA, 5.8S rRNA, LSU-rRNA)	0.00677
**GO:0006284**	base-excision repair	0.00743
**GO:0007095**	mitotic G2 DNA damage checkpoint	0.00873
**GO:0001703**	gastrulation with mouth forming first	0.00884
**GO:0000469**	cleavage involved in rRNA processing	0.009
**GO:0007089**	traversing start control point of mitotic cell cycle	0.00915
**GO:0080034**	host response to induction by symbiont of tumor, nodule or growth in host	0.00915
**GO:0035247**	peptidyl-arginine omega-N-methylation	0.00915
**GO:0000470**	maturation of LSU-rRNA	0.00915
**GO:0009219**	pyrimidine deoxyribonucleotide metabolic process	0.00915
**GO:0031122**	cytoplasmic microtubule organization	0.00965
**GO:0040022**	feminization of hermaphroditic germ-line	0.00965
**GO:0006607**	NLS-bearing protein import into nucleus	0.00965
**GO:0000447**	endonucleolytic cleavage in ITS1 to separate SSU-rRNA from 5.8S rRNA and LSU-rRNA from tricistronic rF	0.00965
**GO:0000460**	maturation of 5.8S rRNA	0.00997
	**Cluster 2**	
**GO:1901671**	positive regulation of superoxide dismutase activity	0.0018
**GO:0048318**	axial mesoderm development	0.0018
**GO:0034463**	90S preribosome assembly	0.0018
**GO:0042474**	middle ear morphogenesis	0.0073
**GO:0006412**	translation	0.0075
	**Cluster 3**	
**GO:0033120**	positive regulation of RNA splicing	0.00044
**GO:0000162**	tryptophan biosynthetic process	0.00087
**GO:0006777**	Mo-molybdopterin cofactor biosynthetic process	0.00144
**GO:0034508**	centromere complex assembly	0.00144
**GO:0032324**	molybdopterin cofactor biosynthetic process	0.00298
**GO:0045144**	meiotic sister chromatid segregation	0.00298
**GO:0006913**	nucleocytoplasmic transport	0.0074
	**Cluster 4**	
**GO:0006145**	purine nucleobase catabolic process	0,0011
**GO:0006573**	valine metabolic process	0,0024
**GO:0042430**	indole-containing compound metabolic process	0,003

**GO:0031365**	N-terminal protein amino acid modification	0,004
**GO:0008593**	regulation of Notch signaling pathway	0,0041
**GO:0055114**	oxidation-reduction process	0,0041
**GO:0009083**	branched-chain amino acid catabolic process	0,0072
**GO:0009072**	aromatic amino acid family metabolic process	0,0085
	**Cluster 5**	
**GO:0006891**	intra-Golgi vesicle-mediated transport	6.1e-05
**GO:0019915**	lipid storage	0.00028
**GO:0046474**	glycerophospholipid biosynthetic process	0.00046
**GO:0051208**	sequestering of calcium ion	9,00E-04
**GO:0006890**	retrograde vesicle-mediated transport, Golgi to endoplasmic reticulum	0.00115
**GO:0048205**	COPI coating of Golgi vesicle	0.00116
**GO:0007030**	Golgi organization	0.00126
**GO:0016185**	synaptic vesicle budding from presynaptic endocytic zone membrane	0.00239
**GO:0035158**	regulation of tube diameter, open tracheal system	0.00241
**GO:0044106**	cellular amine metabolic process	0.00269
**GO:0019884**	antigen processing and presentation of exogenous antigen	0.00289
**GO:0010883**	regulation of lipid storage	0.00321
**GO:0006901**	vesicle coating	0.0039
**GO:0002474**	antigen processing and presentation of peptide antigen via MHC class I	0.0042
**GO:0030536**	larval feeding behavior	0.00472
**GO:0046487**	glyoxylate metabolic process	0.00472
**GO:0035149**	lumen formation, open tracheal system	0.00472
**GO:0072578**	neurotransmitter-gated ion channel clustering	0.00472
**GO:0048079**	regulation of cuticle pigmentation	0.00472
**GO:0032075**	positive regulation of nuclease activity	0.00511
**GO:0019886**	antigen processing and presentation of exogenous peptide antigen via MHC class II	0.00511
**GO:0015908**	fatty acid transport	0.00534
**GO:0030258**	lipid modification	0.00753
**GO:0050690**	regulation of defense response to virus by virus	0.00772
**GO:0034629**	cellular protein-containing complex localization	0.00772
**GO:0007286**	spermatid development	0.00775
**GO:0045807**	positive regulation of endocytosis	0.00775
**GO:0032101**	regulation of response to external stimulus	0.00936
**GO:0044070**	regulation of anion transport	0.00983
	**Cluster 6**	
**GO:0008360**	regulation of cell shape	1.7e-05
**GO:0061024**	membrane organization	3.4e-05
**GO:0019226**	transmission of nerve impulse	4.6e-05
**GO:0016197**	endosomal transport	4.6e-05
**GO:0030833**	regulation of actin filament polymerization	5.5e-05
**GO:0048011**	neurotrophin TRK receptor signaling pathway	5.5e-05
**GO:0008543**	fibroblast growth factor receptor signaling pathway	0.00013
**GO:0060341**	regulation of cellular localization	0.00013
**GO:0019228**	neuronal action potential	0.00016
**GO:0007015**	actin filament organization	2,00E-04
**GO:0007173**	epidermal growth factor receptor signaling pathway	0.00021
**GO:0030163**	protein catabolic process	0.00021

**GO:0006821**	chloride transport	0.00024
**GO:0035023**	regulation of Rho protein signal transduction	0.00028
**GO:0043547**	positive regulation of GTPase activity	0.00031
**GO:0007110**	meiosis I cytokinesis	0.00034
**GO:0007111**	meiosis II cytokinesis	0.00034
**GO:0016080**	synaptic vesicle targeting	0.00035
**GO:0042552**	myelination	0.00052
**GO:0007268**	chemical synaptic transmission	0.00053
**GO:0033121**	regulation of purine nucleotide catabolic process	0.00055
**GO:0006928**	movement of cell or subcellular component	6,00E-04
**GO:1901615**	organic hydroxy compound metabolic process	6,00E-04
**GO:0051650**	establishment of vesicle localization	0.00062
**GO:0009118**	regulation of nucleoside metabolic process	0.00065
**GO:0006874**	cellular calcium ion homeostasis	0.00074
**GO:0007265**	Ras protein signal transduction	0.00078
**GO:0070588**	calcium ion transmembrane transport	0.00083
**GO:0032147**	activation of protein kinase activity	0.00085
**GO:0019886**	antigen processing and presentation of exogenous peptide antigen via MHC class II	0.00095
**GO:0048259**	regulation of receptor-mediated endocytosis	0.00096
**GO:0044089**	positive regulation of cellular component biogenesis	0.00098
**GO:0007409**	axonogenesis	0.00113
**GO:0051046**	regulation of secretion	0.0012
**GO:0030155**	regulation of cell adhesion	0.00123
**GO:0034314**	Arp2/3 complex-mediated actin nucleation	0.00151
**GO:0031018**	endocrine pancreas development	0.00151
**GO:0007031**	peroxisome organization	0.00155
**GO:0071242**	cellular response to ammonium ion	0.00155
**GO:0048193**	Golgi vesicle transport	0.00164
**GO:0048137**	spermatocyte division	0.0018
**GO:0048814**	regulation of dendrite morphogenesis	0.0018
**GO:0006487**	protein N-linked glycosylation	0.00187
**GO:0030031**	cell projection assembly	0.0019
**GO:0030855**	epithelial cell differentiation	0.00194
**GO:0000212**	meiotic spindle organization	0.00198
**GO:0000281**	mitotic cytokinesis	0.00206
**GO:0006635**	fatty acid beta-oxidation	0.00206
**GO:0042981**	regulation of apoptotic process	0.00207
**GO:0043161**	proteasome-mediated ubiquitin-dependent protein catabolic process	0.00215
**GO:0048666**	neuron development	0.00219
**GO:0046488**	phosphatidylinositol metabolic process	0.00224
**GO:0010565**	regulation of cellular ketone metabolic process	0.00238
**GO:0009968**	negative regulation of signal transduction	0.00239
**GO:0030242**	autophagy of peroxisome	0.0025
**GO:0051278**	fungal-type cell wall polysaccharide biosynthetic process	0.0025
**GO:0046325**	negative regulation of glucose import	0.0025
**GO:0035372**	protein localization to microtubule	0.0025
**GO:0060049**	regulation of protein glycosylation	0.0025
**GO:0016137**	glycoside metabolic process	0.0025

**GO:0035118**	embryonic pectoral fin morphogenesis	0.0025
**GO:0010256**	endomembrane system organization	0.00252
**GO:0032880**	regulation of protein localization	0.00261
**GO:0009617**	response to bacterium	0.00273
**GO:0050654**	chondroitin sulfate proteoglycan metabolic process	0.00275
**GO:0001707**	mesoderm formation	0.00275
**GO:1900544**	positive regulation of purine nucleotide metabolic process	0.00275
**GO:0051261**	protein depolymerization	0.00291
**GO:0007605**	sensory perception of sound	0.00291
**GO:0032506**	cytokinetic process	0.00294
**GO:0006023**	aminoglycan biosynthetic process	0.00311
**GO:0051289**	protein homotetramerization	0.00311
**GO:0051351**	positive regulation of ligase activity	0.00331
**GO:0051443**	positive regulation of ubiquitin-protein transferase activity	0.00331
**GO:0007626**	locomotory behavior	0.00351
**GO:0030010**	establishment of cell polarity	0.00356
**GO:0007112**	male meiosis cytokinesis	0.00359
**GO:0046135**	pyrimidine nucleoside catabolic process	0.00359
**GO:0030166**	proteoglycan biosynthetic process	0.00359
**GO:0019932**	second-messenger-mediated signaling	0.00391
**GO:0002479**	antigen processing and presentation of exogenous peptide antigen via MHC class I, TAP-dependent	0.00394
**GO:0006893**	Golgi to plasma membrane transport	0.00401
**GO:0005979**	regulation of glycogen biosynthetic process	0.00405
**GO:0010677**	negative regulation of cellular carbohydrate metabolic process	0.00405
**GO:0031340**	positive regulation of vesicle fusion	0.00405
**GO:0072329**	monocarboxylic acid catabolic process	0.00413
**GO:0023056**	positive regulation of signaling	0.00424
**GO:0051130**	positive regulation of cellular component organization	0.00433
**GO:0007254**	JNK cascade	0.00434
**GO:0030203**	glycosaminoglycan metabolic process	0.00444
**GO:0051592**	response to calcium ion	0.00453
**GO:0007188**	adenylate cyclase-modulating G protein-coupled receptor signaling pathway	0.00469
**GO:0032940**	secretion by cell	0.00501
**GO:0050730**	regulation of peptidyl-tyrosine phosphorylation	0.00506
**GO:0043406**	positive regulation of MAP kinase activity	0.00506
**GO:0090130**	tissue migration	0.00513
**GO:0030433**	ubiquitin-dependent ERAD pathway	0.00534
**GO:0008356**	asymmetric cell division	0.00541
**GO:0051653**	spindle localization	0.00541
**GO:0034620**	cellular response to unfolded protein	0.00597
**GO:0006977**	DNA damage response, signal transduction by p53 class mediator resulting in cell cycle arrest	0.00605
**GO:0050679**	positive regulation of epithelial cell proliferation	0.00616
**GO:0120035**	regulation of plasma membrane bounded cell projection organization	0.00617
**GO:0035020**	regulation of Rac protein signal transduction	0.00639
**GO:0006895**	Golgi to endosome transport	0.00639
**GO:0007608**	sensory perception of smell	0.00639
**GO:0006687**	glycosphingolipid metabolic process	0.00639
**GO:0051352**	negative regulation of ligase activity	0.00642

**GO:0051444**	negative regulation of ubiquitin-protein transferase activity	0.00642
**GO:0006904**	vesicle docking involved in exocytosis	0.00679
**GO:0050851**	antigen receptor-mediated signaling pathway	0.00679
**GO:0019933**	cAMP-mediated signaling	0.00679
**GO:0000916**	actomyosin contractile ring contraction	0.00679
**GO:0044273**	sulfur compound catabolic process	0.00702
**GO:0001745**	compound eye morphogenesis	0.00713
**GO:0007596**	blood coagulation	0.00741
**GO:0005975**	carbohydrate metabolic process	0.0076
**GO:0043062**	extracellular structure organization	0.00778
**GO:0061640**	cytoskeleton-dependent cytokinesis	0.00794
**GO:0010647**	positive regulation of cell communication	0.00805
**GO:0001505**	regulation of neurotransmitter levels	0.00823
**GO:0044262**	cellular carbohydrate metabolic process	0.00833
**GO:1904062**	regulation of cation transmembrane transport	0.00835
**GO:0045921**	positive regulation of exocytosis	0.00843
**GO:0002474**	antigen processing and presentation of peptide antigen via MHC class I	0.00868
**GO:0070207**	protein homotrimerization	0.009
**GO:0071377**	cellular response to glucagon stimulus	0.009
**GO:0019673**	GDP-mannose metabolic process	0.009
**GO:0075306**	regulation of conidium formation	0.009
**GO:0007144**	female meiosis I	0.009
**GO:0002673**	regulation of acute inflammatory response	0.009
**GO:0044091**	membrane biogenesis	0.009
**GO:0030866**	cortical actin cytoskeleton organization	0.00906
**GO:0030258**	lipid modification	0.00916
**GO:0006984**	ER-nucleus signaling pathway	0.00921
**GO:0048599**	oocyte development	0.00929
**GO:0048488**	synaptic vesicle endocytosis	0.00931
**GO:0090288**	negative regulation of cellular response to growth factor stimulus	0.00951
**GO:0010863**	positive regulation of phospholipase C activity	0.00951
**GO:0001667**	ameboidal-type cell migration	0.00972
**GO:0097164**	ammonium ion metabolic process	0.00983
**GO:0001933**	negative regulation of protein phosphorylation	0.00983
**GO:0046879**	hormone secretion	0.00997
	**Cluster 7**	
**GO:0008045**	motor neuron axon guidance	2.6e-09
**GO:0033121**	regulation of purine nucleotide catabolic process	3.9e-07
**GO:0009118**	regulation of nucleoside metabolic process	4.4e-07
**GO:0072015**	glomerular visceral epithelial cell development	4.2e-05
**GO:0003382**	epithelial cell morphogenesis	6.6e-05
**GO:0007155**	cell adhesion	8.4e-05
**GO:0031578**	mitotic spindle orientation checkpoint	1,00E-04
**GO:0001736**	establishment of planar polarity	0.00012
**GO:0050680**	negative regulation of epithelial cell proliferation	0.00012
**GO:0043547**	positive regulation of GTPase activity	0.00014
**GO:0045571**	negative regulation of imaginal disc growth	0.00019
**GO:0071260**	cellular response to mechanical stimulus	0.00019

**GO:0042491**	inner ear auditory receptor cell differentiation	2,00E-04
**GO:0019991**	septate junction assembly	0.00026
**GO:0043588**	skin development	0.00031
**GO:0007160**	cell-matrix adhesion	0.00037
**GO:0016321**	female meiosis chromosome segregation	0.00043
**GO:0030334**	regulation of cell migration	0.00044
**GO:0007136**	meiotic prophase II	0.00049
**GO:0045061**	thymic T cell selection	0.00049
**GO:2000311**	regulation of AMPA receptor activity	0.00049
**GO:0033563**	dorsal/ventral axon guidance	0.00055
**GO:0000916**	actomyosin contractile ring contraction	0.00055
**GO:0030029**	actin filament-based process	0.00063
**GO:0019954**	asexual reproduction	0.00067
**GO:0042048**	olfactory behavior	8,00E-04
**GO:0090529**	cell septum assembly	8,00E-04
**GO:0002286**	T cell activation involved in immune response	8,00E-04
**GO:0035120**	post-embryonic appendage morphogenesis	0.00087
**GO:0035114**	imaginal disc-derived appendage morphogenesis	0.00095
**GO:2000146**	negative regulation of cell motility	0.00106
**GO:0043087**	regulation of GTPase activity	0.00121
**GO:0043542**	endothelial cell migration	0.00134
**GO:0061245**	establishment or maintenance of bipolar cell polarity	0.00134
**GO:0008104**	protein localization	0.00139
**GO:0070863**	positive regulation of protein exit from endoplasmic reticulum	0.00144
**GO:0033599**	regulation of mammary gland epithelial cell proliferation	0.00144
**GO:0032232**	negative regulation of actin filament bundle assembly	0.00144
**GO:1900006**	positive regulation of dendrite development	0.00144
**GO:0051764**	actin crosslink formation	0.00144
**GO:0000281**	mitotic cytokinesis	0.00145
**GO:0071478**	cellular response to radiation	0.00176
**GO:0003008**	system process	0.00189
**GO:0060070**	canonical Wnt signaling pathway	0.002
**GO:1905330**	regulation of morphogenesis of an epithelium	0.00201
**GO:0030856**	regulation of epithelial cell differentiation	0.00201
**GO:0048570**	notochord morphogenesis	0.00201
**GO:0043113**	receptor clustering	0.00201
**GO:0001525**	angiogenesis	0.00209
**GO:0006928**	movement of cell or subcellular component	0.00229
**GO:0001935**	endothelial cell proliferation	0.00233
**GO:0007391**	dorsal closure	0.00233
**GO:0007560**	imaginal disc morphogenesis	0.00252
**GO:0051493**	regulation of cytoskeleton organization	0.00254
**GO:0001764**	neuron migration	0.00257
**GO:0031100**	animal organ regeneration	0.00257
**GO:0006468**	protein phosphorylation	0.00259
**GO:2000114**	regulation of establishment of cell polarity	0.00284
**GO:0032489**	regulation of Cdc42 protein signal transduction	0.00284
**GO:0030835**	negative regulation of actin filament depolymerization	0.00284

**GO:0051971**	positive regulation of transmission of nerve impulse	0.00284
**GO:0010091**	trichome branching	0.00284
**GO:0003094**	glomerular filtration	0.00284
**GO:0048646**	anatomical structure formation involved in morphogenesis	0.00307
**GO:0051056**	regulation of small GTPase mediated signal transduction	0.0032
**GO:0046847**	filopodium assembly	0.00322
**GO:0035220**	wing disc development	0.00349
**GO:0035023**	regulation of Rho protein signal transduction	0.00367
**GO:0007264**	small GTPase mediated signal transduction	0.00391
**GO:0034333**	adherens junction assembly	0.00399
**GO:2000027**	regulation of animal organ morphogenesis	0.00399
**GO:0060562**	epithelial tube morphogenesis	0.00443
**GO:0030582**	reproductive fruiting body development	0.00462
**GO:0030708**	germarium-derived female germ-line cyst encapsulation	0.00466
**GO:0048846**	axon extension involved in axon guidance	0.00466
**GO:0045730**	respiratory burst	0.00466
**GO:0035204**	negative regulation of lamellocyte differentiation	0.00466
**GO:0008302**	female germline ring canal formation, actin assembly	0.00466
**GO:0050665**	hydrogen peroxide biosynthetic process	0.00466
**GO:0042554**	superoxide anion generation	0.00466
**GO:0036360**	sorocarp stalk morphogenesis	0.00466
**GO:1901069**	guanosine-containing compound catabolic process	0.00519
**GO:0008360**	regulation of cell shape	0.00519
**GO:0040039**	inductive cell migration	0.00566
**GO:0009887**	animal organ morphogenesis	0.00571
**GO:0007009**	plasma membrane organization	0.00573
**GO:0001838**	embryonic epithelial tube formation	0.00573
**GO:0051049**	regulation of transport	0.00613
**GO:0050764**	regulation of phagocytosis	0.00677
**GO:0051026**	chiasma assembly	0.00689
**GO:0000915**	actomyosin contractile ring assembly	0.00689
**GO:0050806**	positive regulation of synaptic transmission	0.00689
**GO:0032510**	endosome to lysosome transport via multivesicular body sorting pathway	0.00689
**GO:0046755**	viral budding	0.00689
**GO:0003081**	regulation of systemic arterial blood pressure by renin-angiotensin	0.00689
**GO:0048812**	neuron projection morphogenesis	0.0069
**GO:0042060**	wound healing	0.00693
**GO:0007265**	Ras protein signal transduction	0.00709
**GO:0046039**	GTP metabolic process	0.00762
**GO:0044766**	multi-organism transport	0.00791
**GO:0098609**	cell-cell adhesion	0.0081
**GO:0060538**	skeletal muscle organ development	0.0081
**GO:0010769**	regulation of cell morphogenesis involved in differentiation	0.00854
**GO:0021953**	central nervous system neuron differentiation	0.00916
**GO:0070507**	regulation of microtubule cytoskeleton organization	0.00939
**GO:0072089**	stem cell proliferation	0.00939
**GO:0051645**	Golgi localization	0.00951
**GO:0045887**	positive regulation of synaptic growth at neuromuscular junction	0.00951

**GO:0044130**	negative regulation of growth of symbiont in host	0.00951
**GO:0010596**	negative regulation of endothelial cell migration	0.00951
**GO:0051492**	regulation of stress fiber assembly	0.00951
**GO:0035090**	maintenance of apical/basal cell polarity	0.00951
**GO:0031103**	axon regeneration	0.00951
**GO:0048592**	eye morphogenesis	0.00965
	**Cluster 8**	
**GO:0008285**	negative regulation of cell population proliferation	0.00016
**GO:0007169**	transmembrane receptor protein tyrosine kinase signaling pathway	0.00088
**GO:0007267**	cell-cell signaling	0.00101
**GO:0030336**	negative regulation of cell migration	0.00105
**GO:0034109**	homotypic cell-cell adhesion	0.00183
**GO:0007596**	blood coagulation	0.00194
**GO:0032387**	negative regulation of intracellular transport	0.00259
**GO:0045765**	regulation of angiogenesis	0.00302
**GO:0051612**	negative regulation of serotonin uptake	0.00313
**GO:0007263**	nitric oxide mediated signal transduction	0.00313
**GO:0042738**	exogenous drug catabolic process	0.00313
**GO:0009725**	response to hormone	0.00401
**GO:0051224**	negative regulation of protein transport	0.0045
**GO:0010959**	regulation of metal ion transport	0.00594
**GO:0031284**	positive regulation of guanylate cyclase activity	0.00626
**GO:0034332**	adherens junction organization	0.00657
**GO:0045216**	cell-cell junction organization	0.0069
**GO:0010038**	response to metal ion	0.00858
**GO:0006154**	adenosine catabolic process	0.00937
**GO:0045776**	negative regulation of blood pressure	0.00937
**GO:0045779**	negative regulation of bone resorption	0.00937
**GO:0010761**	fibroblast migration	0.00937
**GO:0009816**	defense response to bacterium, incompatible interaction	0.00937
	**Cluster 9**	
**GO:0015991**	ATP hydrolysis coupled proton transport	0.0015
