# Supplementary material for: A unicellular relative of animals generates a layer of polarized cells by actomyosin-dependent cellularization
Source: eLife. 2019 Oct 28;8:e49801. doi: 10.7554/eLife.49801 (PMC6855841; doi:10.7554/eLife.49801)
Supplement: Figure 5—source data 2. [file elife-49801-fig5-data2.pdf]

Common gene name	Reference gene name
**alpha-tubulin**	Sarc4_g31031T
**beta-tubulin**	Sarc4_g29856T
**Arp2**	Sarc4_g15431T
**Arp3**	Sarc4_g29717T
**Arp4**	Sarc4_g33563T
**Formin1**	Sarc4_g11912T
**Formin2**	Sarc4_g18997T
**Formin3**	Sarc4_g26234T_Sarc4_g26235T
**Formin4**	Sarc4_g26707T
**Formin5**	Sarc4_g32729T
**Formin6**	Sarc4_g8229T
**Septin1**	Sarc4_g12255T
**Septin2**	Sarc4_g19431T
**Septin3**	Sarc4_g20537T
**Septin4**	Sarc4_g8850T
**Myosin II**	Sarc4_g23768T
**Myosin V**	Sarc4_g13387T
**Profilin / Chickadee**	Sarc4_g5267T
**Cofilin / Twinstar**	Sarc4_g6950T
**Integrin alpha**	Sarc4_g1310T
**Integrin beta**	Sarc4_g33805T
**Pinch**	Sarc4_g2938T
**Parvin**	Sarc4_g25286T
**Paxilin**	Sarc4_g20792T
**Talin**	Sarc4_g3704T_Sarc4_g3705T_Sarc4_g3706T
**Vinculin / alpha-catenin**	Sarc4_g11224T
**alpha-actinin**	Sarc4_g4850T
**Aardvark1**	Sarc4_g24897T
**Aardvark2**	Sarc4_g24898T
**Aardvark3**	Sarc4_g33617T
**Kinesin1**	Sarc4_g8678T
**Kinesin2**	Sarc4_g14701T
**Kinesin3**	Sarc4_g23312T

**Kinesin4**	Sarc4_g23709T_Sarc4_g23711T_Sarc4_g23712T
**Kinesin5**	Sarc4_g23709T_Sarc4_g23711T_Sarc4_g23712T
**Kinesin6**	Sarc4_g27337T
**Kinesin7**	Sarc4_g29337T
**Kinesin8**	Sarc4_g29515T
**Kinesin9**	Sarc4_g31878T
**Kinesin10**	Sarc4_g8472T_Sarc4_g8473T
**Kinesin11**	Sarc4_g8472T_Sarc4_g8473T
**Rho1**	Sarc4_g28650T
**Rho2**	Sarc4_g29101T
**Rho3**	Sarc4_g32829T
**Rho4**	Sarc4_g5839T
**Rab11_1**	Sarc4_g17148T
**Rab11_2**	Sarc4_g2031T
**Rab5_1**	Sarc4_g1679T
**Rab5_2**	Sarc4_g14741T
